# Scale-up disaggregation of palygorskite crystal bundles via ultrasonic process for using as potential drilling fluid

**DOI:** 10.1016/j.ultsonch.2022.106128

**Published:** 2022-08-19

**Authors:** Jiang Xu, Wenbo Wang, Yushen Lu, Hong Zhang, Yuru Kang, Bin Mu, Ye Qian, Aiqin Wang

**Affiliations:** a Key Laboratory of Clay Mineral Applied Research of Gansu Province, Center of Eco-material and Green Chemistry, Lanzhou Institute of Chemical Physics, Chinese Academy of Sciences, Lanzhou 730000, PR China; b College of Chemistry and Chemical Engineering, Inner Mongolia University, Hohhot 010021, PR China; c Center of Materials Science and Optoelectronics Engineering, University of Chinese Academy of Sciences, Beijing 100049, PR China; d Department of Civil Engineering, The University of Hong Kong, Pokfulam Road, Hong Kong, PR China

**Keywords:** Palygorskite, Disaggregation, Ultrasound, Rod-like crystal, Drilling fluid

## Abstract

High-efficient disaggregation of palygorskite (PAL) crystal aggregates into individual nanorods is the key to exploiting its nanometer properties, which remains a challenge at present. The sonochemical cavitation effects have been successfully employed for the intensification of physical and chemical processing applications, but it still lacks the relevant study on the scale-up disaggregation of PAL crystal bundles. Here, the energy-efficient, scale-up ultrasonic process was developed to disaggregate PAL aggregates in batches, and the effects of ultrasonic treatment time, temperature, and power on physicochemical features of PAL were systematically investigated. The results showed that the single dispersed PAL nanorods could be continuously produced by sonicating 15 wt% of PAL suspension at 20 kHz, 2000 W and 30 °C for 5 min retaining the original nanorod length and layered-chain structure. It also greatly improved the dispersion of nanorod crystal, specific surface area and suspension stability of PAL. The ultrasonically disaggregated PAL has a higher pulping rate in water (14.96 m^3^/t) and saturated NaCl system (14.45 m^3^/t), which is significantly better than that of natural PAL in water (14.72 m^3^/t) and saturated NaCl solution (12.37 m^3^/t). It suggests that the disaggregated PAL exhibits excellent potential and adaptability as a viscosity enhancer for drilling fluid. Therefore, this work provides a feasible and efficient ultrasonic process for large-scale industrialized disaggregation of PAL crystal bundles, laying a foundation for the high-value utilization of natural PAL as one-dimensional nanomaterials.

## Highlights

• Ultrasonic process was used to large-scale disaggregate PAL crystal bundles.
• Single dispersed PAL nanorods were produced by sonication at 20 kHz, 2000 W and 30 °C for 5 min.
• The dispersion and suspension stability of disaggregated PAL were greatly improved.
• The pulping rates of disaggregated PAL in water and brine system are ≥ 14.45 m^3^/t.

## Graphical abstract

**Figure:**
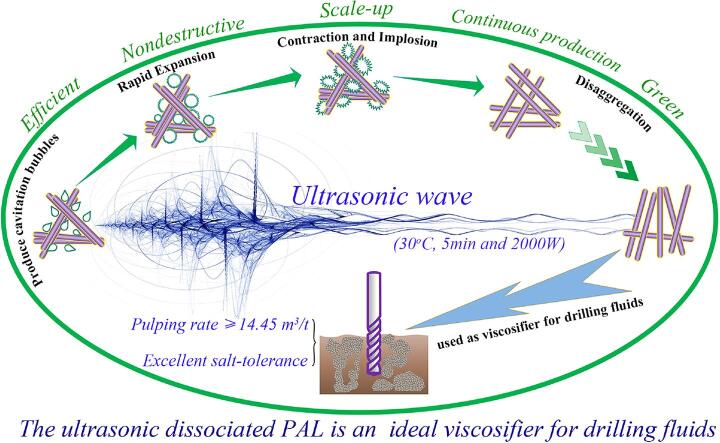

## 1 Introduction

Palygorskite (PAL) is a kind of natural hydrated magnesium aluminum silicate clay mineral with unique nanorod crystal morphology [1], [2], which has been widely used in many fields such as oil decoloration [3], organic-inorganic hybrid pigments [4], drilling mud [5], carrier of catalysts [6], polymer composites [7] and wastewater treatment [8]. However, the nanorods of natural PAL tend to exist as aggregates or crystal bundles due to the hydrogen bonding, Van der Waals' force and electrostatic interaction, and it is difficult to exploit the unique nanomaterial properties of PAL for constructing functional materials [9], [10], [11]. Therefore, it is indispensable to disaggregate the bulk PAL aggregates or crystal bundles into individual nanorods for the high-value utilization of natural PAL.

Mechanical treatment in the dry state (*e.g.* squeeze, ball milling, and grinding) or in the wet state (*e.g.* high-speed shearing) have been used to disaggregate PAL crystal bundles [12], [13], [14]. The dry treatment with a strong or multiple mechanical shear process easily leads to the fracture of rod crystals or the transformation of crystal structure into amorphous one [12], [15], [16], while the wet treatment requires a high shear rate and long time, which only partially splits the crystal bundles [17]. In previous studies, the high-pressure homogenization technology was developed for effective disaggregation of the PAL crystal bundles with the help of strong shear force and cavitation effects during the instantaneous pressurization and pressure-relief process [18], [19]. However, the high-pressure homogenization process is difficult to realize the large-scale continuous production due to the special pressurizing and sealing equipment. What’s worse, the residual trace amounts of quartz sand in the PAL slurry still can strongly wear the high-pressure homogenizer even after purification, which results in the decrease in the homogenization pressure and disaggregation efficiency, and even maintenance outages at the expense of a great deal of maintenance cost and production. Therefore, it is an urgent need to develop a feasible industrial technology for large-scale continuous disaggregation of PAL crystal bundles.

Ultrasonic wave is a high frequency (above 20 kHz) and short-wavelength (even a few micrometers) sound wave with good directionality, strong penetrability, and concentrated energy [20], [21]. When ultrasonic wave propagates in a medium, especially in a liquid medium and heterogeneous phases interfaces, a series of spectacular cavitation phenomena occur continuously and locally on a micro-scale, such as extremely high local temperature and pressure, high shear stress near the bubble wall, and micro-jets near the solid surface caused by asymmetric collapse of bubble and turbulence [22], [23], [24]. The ultrasonic process has been widely used in many fields such as cleaning [25], welding [26], fine chemicals, and others [27]. The literature analysis also shows that the ultrasonic waves can obviously reduce the particle size of clay minerals and natural cellulose. For example, Franco *et al.* reported that the particle size of kaolinite samples reduced from 0.1–30 µm to mostly lower than 5 µm after ultrasonic irradiation for 20 h retaining its crystalline structure and lamellar morphology [28]. The particles of cellulose aqueous suspension with a large size of 63 µm was reduced to the nanometer scale by a sequential approach of hydrodynamic cavitation and sonication for 6 h [29]. More notably, the cavitation effect generated in the ultrasonic process also has been employed to disaggregate the PAL crystal bundles, but it is limited to the laboratory scale with reactors holding only a few milliliters of capacity [24]. There are no studies on the large-scale disaggregation of PAL to prepare nanomaterials by ultrasonic waves up to now.

The reaction efficiency of sonochemical reactions is dramatically influenced by the processing time, bulk temperature, acoustic intensity, and the liquid phase physicochemical properties [24]. Due to the strong nonlinear behavior and very large scale-up ratios, it is difficult to effectively operate an industrial-scale sonochemical reactor for a given application (*e.g.* disaggregation of PAL crystal bundles). Therefore, it is required to quantitatively study the relationship between key parameters and product performance [30], especially in scale-up production lines. On the other hand, PAL is commonly used as a viscosifier for drilling fluids while bentonite becomes inoperative [31], and its performance is strongly related to the particle morphology and surface charge of PAL at a relative low solid-phase levels [32]. The best way for high-effective utilization of PAL in drilling fluids is to improve the dispersibility and colloidal properties through a large-scale ultrasonic disaggregation process.

**Scheme 1:**
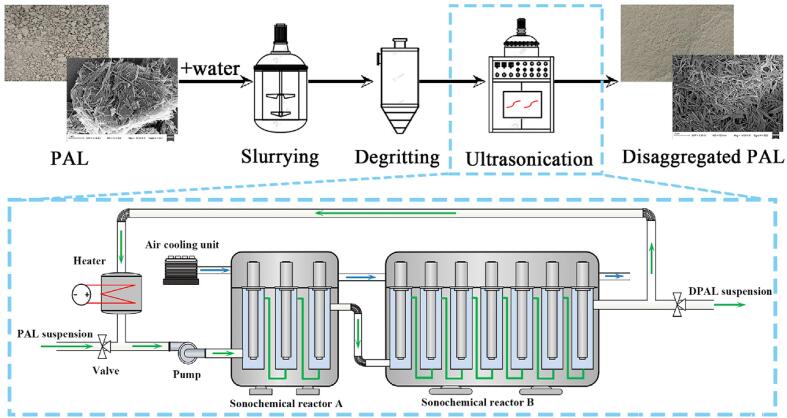
Scale-up disaggregation process of PAL crystal bundles *via* ultrasonic treatment.

Based on the above background, the large-scale disaggregation of natural PAL crystal bundles was performed to produce PAL individual nanorods by industrial ultrasonic process in this study (Scheme 1). The physicochemical characteristics, including morphology, crystal structure, chemical compositions, and specific surface area of the PAL before and after ultrasonic treatment were systematically analyzed and discussed. Furthermore, the potential application of the ultrasonically disaggregated PAL was evaluated as a viscosity enhancer of drilling fluid in water and high salinity environment using the pulping rate as the key technical index.

## 2 Materials and methods

### 2.1 Materials

The raw PAL was taken from the Mingguang mine (Anhui Province, China). The XRF chemical compositions of PAL are SiO_2_ 59.61 wt%, Al_2_O_3_ 9.83 wt%, MgO 10.98 wt%, Fe_2_O_3_ 5.19 wt%, CaO 2.61 wt%, and K_2_O 1.08 wt%. The moisture content of natural PAL was about 13.40 wt%, calculated by the weight difference before and after drying at 105 °C for 2 h. The raw PAL was pretreated by three-roller processing before use to obtain flakes with a thickness of less than 1 mm. The concentrated sulfuric acid (H_2_SO_4_, 98%, Analytical grade) was obtained from Shanghai Sinopharm Chemical Reagent Corporation (Shanghai, China). Sodium chloride (NaCl, Analytical grade) was purchased from Xilong Scientific Corporation (Guangdong Province, China).

### 2.2 Scale-up disaggregation of PAL crystal bundles via ultrasonic process

The scale-up disaggregation of PAL crystal bundles *via* ultrasonic process was shown in Scheme 1. The detailed process was as follows: The raw PAL was first crushed and passed through a 100-mesh screen, and then 340 kg of the pretreated PAL (the actual dry weight was 300 kg) was dispersed into 2000 kg of water to form homogeneous suspension under continuous mechanical stir for 4 h. Next, the concentrated sulfuric acid was added to adjust the pH value of the suspension to be 4.5 ± 0.2 for removal of carbonate impurities. Then the suspension was passed through a hydro-cyclone to remove quartz and insoluble large particles. The collected PAL suspension was subsequently treated with ultrasonic processing equipment (power, 2000 W; frequency, 20 kHz; Xuyi Oubaite Clay Material Co., LTD, Jiangsu, China) for different working conditions. Finally, the disaggregated PAL was centrifuged at 5000 rpm for 10 min, and the obtained solid product was dried at 105 °C for 4 h, ground and passed through a 200-mesh sieve.

As depicted in Scheme 1, the ultrasonic processing equipment consists of two different cylindrical sonochemical reactors (denoted as A and B) in series. The effective working volume of reactor A is 15 L, and three ultrasonic transducers (60 cm in the length and 75 mm in the diameter) with a frequency of 20 kHz and a maximum power of 200 W are installed vertically inside. Similarly, the effective working volume of reactor B is 35 L, and seven transducers same as that of reactor A are arranged vertically inside. In the experiment, about 50 L of PAL suspension was pumped into the ultrasonic processing equipment, and the sonochemical reactions were performed in the annulus space between the ultrasonic transducers and the stainless steel reaction. The ultrasonic transducers were immersed into the liquid phase at a height of 550 mm. To test the effect of ultrasonic temperature on the disaggregation degree, the suspension circulating in the system was heated by an interpolated electric heating tube with a maximum heating power of 300 W with water in the outer jacket of the tank, and the sonication was conducted once the temperature of the suspension was achieved. Furthermore, the heat generated by ultrasonic transducers is carried away by circulating cold air.

The raw PAL was labeled as RPAL, while the purified PAL without carbonate and quartz sand was denoted as PPAL. To systematically investigate the effect of ultrasonic conditions on the disaggregation efficiency, the samples prepared at different ultrasonic time were denoted as PPAL-30-*x* (30 °C, *x* = 2, 5, 8, 10, and 12 min), and the samples obtained at high ultrasonic temperatures were marked as PPAL-50-5 and PPAL-70-5 (50 or 70 °C, 5 min), respectively. In addition, the samples disaggregated only by the sonochemical reactor A (3 × 200 W, 20 kHz) at 30 °C for 5 min were labeled as PPAL-30-5/lp.

### 2.3 Characterizations

The morphology of samples was observed using a field emission scanning electron microscope (FESEM, ULTRA Plus, German) and a transmission electron microscope (TEM, JEM-1200 EX/S, JEOL, Japan). The SEM samples were uniformly dispersed on a copper column and coated with a gold film, while the TEM samples were uniformly dispersed in deionized water and deposited on a copper mesh with a carbon micropore film. The X-ray diffraction (XRD) patterns were performed on a SmartLab SE multifunctional X-ray diffractometer (Rigaku Co., Japan) with a Cu Kα radiation source (40 kV, 40 mA) with the scanning range of 2*θ* = 3–80° by a step of 0.02°/S. The Fourier transform infrared (FTIR) spectra were recorded in the wavenumber range of 4000–400 cm^−1^ on a Nicolet NEXUS FTIR spectrometer (Thermo Nicolet 6700, Thermo Fisher Scientific, USA) using the KBr pellets. The Zeta potential was measured 3 times in parallel on a Malvern Zetasizer Nanosystem (ZEN3600, UK) at 25 °C using a folded capillary to report the average value. Before the test, the powder samples were dispersed in deionized water to form a 0.5% (w/v) of uniform suspension. The particle size measurement was accomplished by measuring the intensity of the scattered light as the laser beam passes through the scattered particle sample [33]. The specific surface areas (S_BET_) and pore structure parameters were determined by an N_2_ adsorption-desorption experiment on an Accelerated Surface Area and Porosimetry System (Micromeritics Instrument Co., USA) at 77 K. The micropore volume (V_micro_) and the micropore area (S_micro_) were calculated by the t-plot method, and the total pore volume (V_total_) and the pore diameter (P_D_) were estimated from the volume of N_2_ adsorbed at a relative pressure P/P_0_ = 0.97 [34]. The chemical composition was assayed on a MiniPal 4 X-ray fluorescence spectrophotometer (PAN analytical Co., Netherlands) by squashing PAL with boric acid powder at a mass ratio of 1:7. The thermal behavior of PAL samples was performed using a simultaneous Thermal Analyzer (STA8000, PerkinElmer, America) in an N_2_ atmosphere (flow rate, 20 mL/min) with a temperature range of 30–800 °C and a heating rate of 10 °C/min.

### 2.4 Measurements of PAL suspension

The rotation viscosity of 7 wt% homogeneous PAL suspension prepared by stirring at 11000 rpm for 20 min was measured at different time intervals (30 s – 10 min) using a rotational viscosimeter (NDJ-8S, Shunyu Hengping Scientific Instrument Co., LTD, Shanghai, China) with 3^#^ spindle at 30 rpm of shear rate. The shear rheological properties were measured on an Anton Paar Physica MCR301 Rheometer (Germany) at 25 °C with a steady shear rate ranging from 0.1 to 200 s^−1^ [33]. The colloidal stability of the PAL suspension was evaluated by the conventional sedimentation technique. Specifically, 2 wt% of uniform suspension obtained at 11000 rpm for 20 min was transferred into a 100 mL scale bucket to directly read the settlement from the graduated cylinder at fixed time intervals. According to the recommended procedures of the OCMA standard and American Petroleum Institute (API) standard [35], the method for measuring the pulping rate was as follows: (i) A series of PAL suspensions with different solid/liquid ratios (40–90 g/L) were prepared by strongly stirred with a high-speed agitator at 11000 rpm for 20 min, then sealed and maintained; (ii) The suspension was stirred again at 11000 rpm for 5 min after standing for 24 h, the viscosity value (*φ*_600_) was measured at the rotor speed of 600 rpm on a rotational viscosimeter (ZNN-D6, Qingdao Haitongda Factory, China), and then the apparent viscosity (*AV*) was calculated according to Eq. (1); (iii) The solid/liquid ratio value corresponding to *AV* = 15 mPa·s (Gw*,* g/L) was back-calculated by the fitting equation after drawing and fitting the curve of *AV* changing with solid/liquid ratio, and then the corresponding pulping rate was calculated (*Q_s_*, m^3^/t) according to Eq. (2) [36], [37]. The slurry was prepared by dispersing RPAL and PPAL-30-5 into deionized water and saturated NaCl solution, respectively, and the samples were labeled as RPAL/PPAL-30-5 + Water and RPAL/PPAL-30-5 + Salt, respectively.

(1) $$\mathrm{AV}=0.5\times \phi_{600}$$

(2) $$\mathrm{Qs}=\;82.3/\left(G_{W}\times 0.1\right)\;+\;0.3$$

## 3 Results and discussion

### 3.1 The changes in micromorphology

**Fig. 1:**
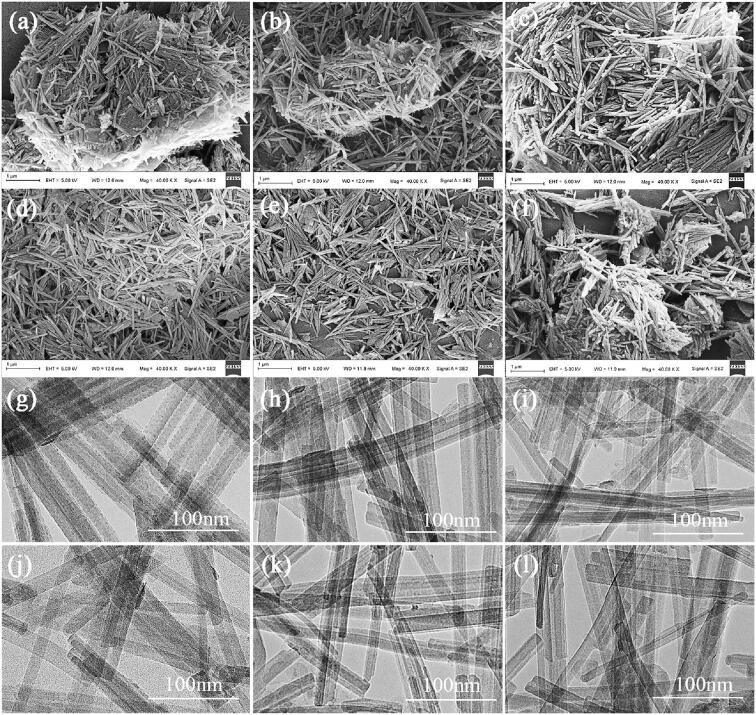
SEM and TEM images of RPAL (a, g), PPAL (b, h), PPAL-30-2 (c, i), PPAL-30-5 (d, j), PPAL-30-12 (e, k) and PPAL-30-5/lp (f, l).

Fig. 1 depicts the SEM and TEM images of RPAL, PPAL, PPAL-30-*x,* and PPAL-30-5/lp. It can be observed from Fig. 1(a and g) that the rod crystals of RPAL are liable to aggregate and form crystal bundles or agglomerations. The size of PPAL aggregates is reduced after treatment with acid, but most of the aggregate clusters still exist (Fig. 1(b and h)), indicating that it is difficult to effectively disaggregate the PAL crystal bundles only using acid treatment. After ultrasonic treatment for 2 min, the aggregates in PPAL-30-2 are further reduced with some crystal bundles (Fig. 1(c and i)). With the increase in the ultrasonic time to 5 min, the dispersion of rod crystals is significantly improved, and more individual nanorods can be observed (Fig. 1(d and j)), indicating that the ultrasonic processing for 5 min can effectively disaggregate the PAL crystal bundles. When millions of microscopic cavitation bubbles implode, the gathered energy is significantly greater than the acoustic energy, which can be rapidly released into the tiny spaces and adjacent surfaces of the PAL crystal bundles in the form of shock waves and high-velocity micro-jets. During this process, the strong impact and shear forces are produced to promote the disaggregation of PAL crystal bundles.

The ultrasonic disaggregation of PAL crystal bundles is a complex multi-step parallel process including the disintegration of inter-bundle connection, the dispersion of stacked crystal bundles, and the disaggregation of crystal bundles into nanorods [38]. Although the irradiation of strong ultrasound (low-frequency and high-power) can bring intense physical effects [24], the degree of disintegration and cavitation effect is insufficient to overwhelm the interactions among the rod crystal bundles, when the ultrasonic treatment time is short (*e.g.* 2 min). Worse still, a fraction of the cavitation energy in the system is dissipated in the disintegration of the inter-bundle connection at the beginning of disaggregation [30]. When the ultrasonic time is extended to 5 min, more tiny spaces and cavitation bubbles are formed, which is conducive to the subsequent implosion of cavitation bubbles to disaggregate the crystal bundles. During this period, the cavitation energy is focused on continuously tearing apart small diameter rod bundles into single crystal rods with the effect of micro-jet (arising from asymmetric bubble collapse). It is worth noting that the use of cylindrical waveguides in a pilot-scale reactor can realize the high-efficient disaggregation of PAL rod bundles, while the processing time drastically reduced to 5 min, thereby reducing the energy consumption of the process [24], [30]. However, the dispersion of PAL crystal bundles is hardly changed after ultrasonic treatment for more than 8 min (Fig. S1 and Fig. 1(e and k)), in which the generated shock waves by the symmetric collapse of cavitation bubbles spread energy evenly to the surrounding environment, while the tearing effect of micro-jet is greatly weakened [39]. In brief, the cavitation mainly works in a transient cavitation mode (severe bubble collapse) at the first 5 min, and then continuously disaggragates PAL crystal bundles in a micro-scale, and transforms into a stable cavitation mode (oscillating bubbles) [30].

The role of heating in ultrasonic disaggregation of PAL crystal bundles was further explored. As shown in Fig. S1, there is no obvious differences in the dispersion of rod crystal in PPAL-50-5 and PPAL-70-5 compared with PPAL-30-5. In fact, the enhanced disintegration can be achieved by the high temperature (up to 5000 K) and high pressure (up to 1000 bar) caused by the ultrasonic energy concentrated on the particle surface [40]. In contrast, the heating is unfavorable for ultrasonic disaggregation of PAL crystal bundles due to the lower cavitational intensity at higher temperature [41]. However, the increase in the temperature of the PAL suspension (50 °C and 70 °C) inevitably leads to an increase in the vapor pressure and viscosity of the liquid, especially for the long rod crystal PAL, both of them can obviously weaken the extent of cavitation effect [42]. Specifically, the vapor content of the cavity increases with increasing liquid vapor pressure, which reduces the energy released during the collapse. The digital photographs of the dissociated suspensions (Fig. S2) also showed that there are more visualized bubbles in PPAL-70-5 of the four kinds of ultrasonic dissociated suspensions even after settling. On the other hand, the heating promotes the evaporation of water and increases the viscosity of the PAL suspension, which also leads to a severe attenuation of the sound intensity and substantial reduction in the active cavitation zone [30].

As detailed in Fig. 1(f and l), most of the clusters of aggregates still exist in PPAL-30-5/lp, the size of the aggregates in PPAL-30-5/lp is close to that of PPAL-30-2, but significantly larger than that of PPAL-30-5. This suggests that the high cavitation intensity significantly increases the number of cavitation events at high power dissipation to promote the efficient disaggregation of PAL crystal bundles [24]. Furthermore, the length distribution of nanorod crystals can be calculated from the SEM image with the software of Image-Pro plus 6.0 (Fig. S3). There is no significant difference in the length of PAL nanorods before and after ultrasonic disaggregation even after treatment at 30 °C for 12 min or 70 °C for 5 min, indicating that the ultrasonic disaggregation is a relatively non-destructive process to the length of PAL nanorod crystal.

### 3.2 The changes in structure and composition

**Fig. 2:**
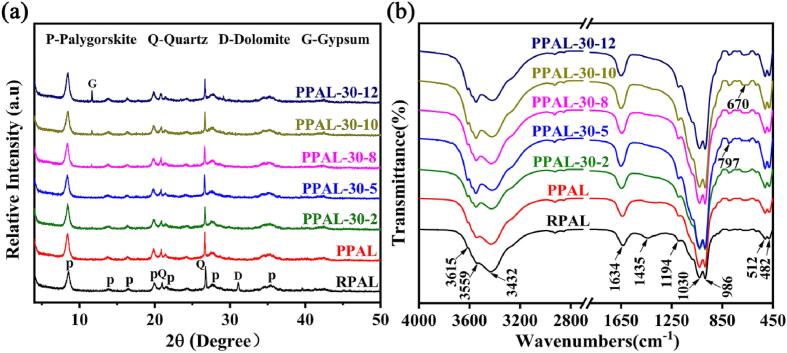
XRD patterns (a) and FTIR spectra (b) of RPAL, PPAL, and PPAL-30-*x.*

The XRD patterns of RPAL, PPAL, and PPAL-30-*x* are illustrated in Fig. 2a. The characteristic peaks of PAL (2*θ* = 8.50°, 13.91°, 16.46°, 19.89°, 21.45°, 27.59°, and 35.44°, JCPDS No. 21–0958), quartz (2*θ* = 20.86° and 26.67°, JCPDS No. 75–0443) and dolomite (2*θ* = 30.94°, JCPDS No. 83–1766) can be observed in the XRD patterns of RPAL [19]. Compared with RPAL, the intensity of diffraction peaks of dolomite and quartz is significantly decreased, indicating that the associated carbonates and quartz sand are partially removed. After further sonication process, the characteristic diffraction peaks of PAL in PPAL-30-*x* remain unchanged, suggesting the crystal structure of PAL is still intact. In addition, the new peak of gypsum (2*θ* = 11.67°, JCPDS No. 74-1905) appears in PPAL-30-8, PPAL-30-10, and PPAL-30-12, indicating that the ultrasonic treatment for longer time promotes the adverse reaction of calcium ion and sulfate acid to form gypsum (Ca^2+^ + SO_4_^2−^ → CaSO_4_) [22]. It is also confirmed by the increased content of SO_3_ in the chemical compositions of PPAL-30-12 (Table S1). The Ca and Mg ions derived from dolomite are dissolved into the solution after acid pretreatment, and the Ca content dropped dramatically from 2.61% for RPAL to 0.49% for PPAL. However, Ca^2+^ and SO_4_^2−^ in the solution fully react to form solid gypsum impurities when the ultrasonic treatment time is extended to 8 min or longer. What’s more, the content of the main elements of PAL (mainly Si, Mg, Al, and Fe) does not change much during the whole ultrasonic treatment, suggesting that ultrasonic treatment has little effect on the chemical compositions of PAL. In a word, the ultrasonic condition of 30 °C and 5 min are optimal for fully disaggregating the PAL rod crystal bundles and preventing the generation of gypsum impurities.

The characteristic absorption bands of PAL are dominant in FTIR spectra (Fig. 2b). The absorption peaks around 3615 and 3559 cm^−1^ are assigned to the O–H stretching vibration of Al-Al-OH and Mg-Fe-OH, respectively [43]. The bands at 3432 and 1634 cm^−1^ correspond to the H-O–H stretching vibration and bending vibration of zeolite water. The absorption peaks near 1194 and 1030 cm^−1^ are assigned to the antisymmetric stretching vibrations of Si-O-Si connecting adjacent two inverted tetrahedrons and Si-O-Si stretching vibration of tetrahedrons. The absorption band at 983 cm^−1^ is ascribed to the stretching vibration of Si-O-Mg, and the band at 797 cm^−1^ corresponds to the Si-O-Si symmetric stretching vibration of quartz. The bands at 512 and 482 cm^−1^ are assigned to the stretching vibration of Si-O-Si and O-Si-O groups in tetrahedron [34]. More importantly, all of these absorption peaks corresponding to the silicon-oxygen tetrahedral, metal-oxygen octahedral, and water molecules in PAL crystals have no differences in RPAL, PPAL, and PPAL-30-*x*. It indicates that the layered-chain structure of PAL is not broken during ultrasonic disaggregation. Meanwhile, the absorption peak belonging to carbonate at 1435 cm^−1^ disappears in PPAL and PPAL-30-*x*, indicating the dissolution of dolomite [33]*.* The absorption peak of sulfate is observed at 678 cm^−1^, which confirms the formation of gypsum [44]. In addition, the mineral compositions and functional groups of PPAL-50/70-5 and RPAL are also almost identical (Fig. S4), suggesting that there is no obvious change for the crystal structure and chemical compositions of PAL with increasing sonication temperature. As shown in Table S1 and Fig. S4, the mineral/chemical compositions and functional groups of PPAL-30-5/lp are almost the same as those of RPAL, which also confirms that both of low-power and high-power ultrasound treatment are the processes of physical disaggregation without changing the layered-chain structure and compositions of PAL.

**Fig. 3:**
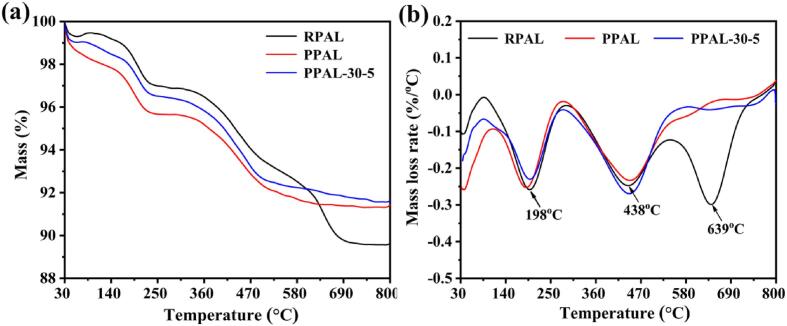
TGA (a) and DTG (b) curves of RPAL, PPAL, and PPAL-30-5.

The channel structure and internal water molecules (such as zeolite water and crystal water) of PAL are crucial for the construction of organic-inorganic hybrid materials [4]. The thermal decomposition behavior of PAL is often used to study the removal process of water molecules and the changes in crystal structure. RPAL, PPAL, and PPAL-30-5 have almost the same weight loss behavior in the region of 30 to 600 °C (Fig. 3), and all of them show the typical weight loss processes of PAL: The elimination of surface adsorbed water and zeolite water below 198 °C; the release of most of crystal water at 200–438 °C; and the removal of remaining crystal water and most of structure water at 438 °C – 600 °C [45]. After ultrasonic disaggregation, the four water molecules of PAL still remain and the channel structure is intact. By contrast, the significant differences in TGA and DTG curves of RPAL occur in the last stage (630–800 °C), which may be due to the decomposition of carbonates at near 639 °C (Fig. 3b) [45].

### 3.3 The changes in surface charge and particle size

**Fig. 4:**
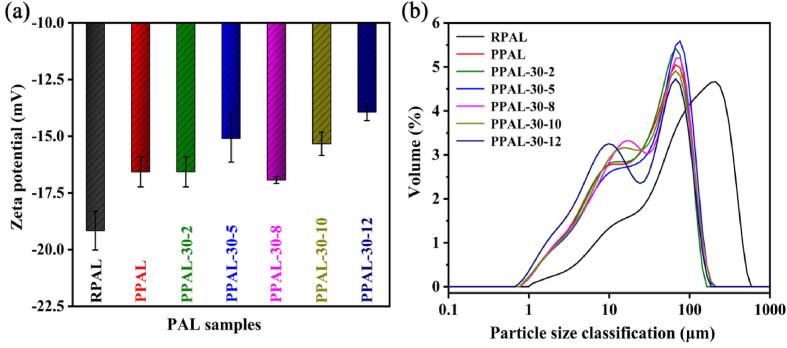
(a) Zeta potential and (b) particle size distribution of RPAL, PPAL, and PPAL-30-*x.*

The surface charge of PAL is mainly derived from the isomorphism substitution and the surface reaction (caused by the pH change of the solution) [46], which may affect the electrostatic interaction between particles, the dispersion and stability of the colloidal solution. Zeta potential is recognized as the common indicator to characterize the surface charge of minerals [47]. As depicted in Fig. 4a, the negative Zeta potential of PPAL decreases from −19.2 mV (RPAL) to −16.5 mV after acid pretreatment, and further decreases after ultrasonic disaggregation (PPAL-30-*x*). It suggests that the hydroxyl group (R-OH) on the surface of PAL rod crystal, especially on the outer surface, may react with hydrogen ions (H^+^) to form R-OH_2_^+^ [47]. PPAL-30*-x* has better dispersion (verified in Fig. 1) and expose more active hydroxyl groups on the surface, which are more conducive to protonation, resulting in a slight reduction in electro-negativity. In our previous study, the Zeta potential of PAL changed from −15.67 mV to −16.27 mV by disaggregation using high-pressure homogenization at 30 MPa followed by modification with phytic acid, and the difference in surface charge was only −0.60 mV [48]. In this study, the change in the surface charge is as high as +2.7 mV due to the ultrasonic disaggregation, which still remained at +1.5 mV even after subtracting the change caused by the solution pH change taken into account of the differences between RPAL and PPAL. It implied that PAL disaggregation and modification using industrial-scale ultrasound equipment may yield the unexpected results due to microscopic changes in surface charge and nano-scale reaction space.

The particle size distribution of RPAL, PPAL, and PPAL-30-*x* is shown in Fig. 4b and Table S2. The D_50_ and D_90_ of RPAL are 91.70 ± 2.60 and 297.33 ± 4.93 μm, respectively, which are reduced to 1/4–1/3 for PPAL and PPAL-30-*x*, indicating that the rod crystal bundles are disaggregated and the dispersion of PAL is greatly improved. Notably, PPAL-30-5 presents the smallest D_90_ (93.23 ± 1.65 μm), showing good dispersion without of gypsum impurities. The increase in the sonication time resulted in a slight increase in the particle size of the disaggregated samples, which may be related to the formation of gypsum impurities, causing the secondary agglomeration of a few nanorods. It was reported that squeezed, frozen, and homogenized can also reduce the diameter distribution of natural PAL from 3.0–4.3 μm to 1.8 μm [33], but the ultrasonic disaggregation allows the continuous industrial production.

### 3.4 The changes in surface textural properties

**Fig. 5:**
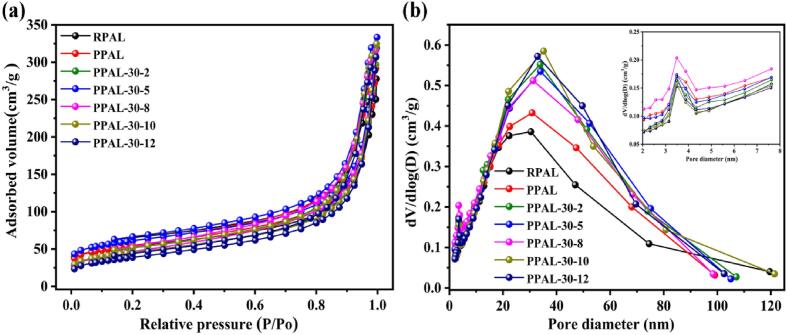
N_2_ adsorption-desorption isotherms (a) and pore size distribution curves (b) of RPAL, PPAL, and PPAL-30-*x.*

Several surface textural parameters, including specific surface area (S_BET_), micropore surface area (S_micro_), micropore volume (V_micro_), external surface area (S_ext_) and average pore diameter (P_D_) can be quantitatively calculated by the N_2_ adsorption-desorption isotherm [1]. The N_2_ adsorption volume of RPAL, PPAL, and PPAL-30-*x* increases significantly under higher relative pressures (P/P_0_ > 0.4) (Fig. 5a), indicating the presence of mesopore and macropore in all samples [49]. The pore size distribution of RPAL, PPAL, and PPAL-30-*x* (Fig. 5b) show two peaks, including a small peak at about 3.5 nm (the inset of Fig. 5b), and a larger peak in the range of 20–100 nm.

In addition, the S_BET_ and pore structure parameters of the samples are summarized in Table 1. S_BET_ increases slightly from 186.40 m^2^/g (RPAL) to 192.44 m^2^/g (PPAL), which may be attributed to the increased PAL purity due to the removal of carbonate [50]. With the prolongation of ultrasonic treatment time, S_BET_ shows a wavy trend of first decreasing, then increasing, and finally decreasing, and the maximum value was obtained in the PPAL-30-5 sample. By contrast, the ultrasonic cavitation significantly improves the dispersion of rod crystals in PPAL-30-5 (D90 is the minimum, Table S2) and induces to form a large number of stacked pores with a small diameter (P_D_ = 6.82 nm) (Fig. 1d and e), resulting in an obvious increase in the values of S_micro_ and V_micro_. However, the increase in the ultrasonic treatment time leads to the formation of gypsum, which may block the stacked pores and induce secondary agglomeration of the disaggregated nanorods, which causes a decrease in the values of S_micro_ and V_micro_. In general, the PPAL-30-5 has the largest S_BET_, S_micro_, and V_micro_, and a large number of small-sized pores, all of which are conducive to constructing functional composites [50].

**Table 1:** Specific surface area and pore structure parameters of PAL and treated samples.

Samples	S_BET_ (m^2^/g)	S_micro_ (m^2^/g)	S_ext_ (m^2^/g)	V_total_ (cm^3^/g)	V_micro_ (cm^3^/g)	P_D_ (nm)
RPAL	186.40	73.40	113.00	0.3135	0.0334	6.73
PPAL	192.44	57.98	134.46	0.3301	0.0259	6.96
PPAL-30-2	160.51	35.17	125.34	0.3393	0.0152	8.45
PPAL-30-5	213.85	77.24	136.60	0.3647	0.0349	6.82
PPAL-30-8	167.88	14.62	153.26	0.3465	0.0050	8.26
PPAL-30-10	161.46	38.32	123.14	0.3435	0.0167	8.51
PPAL-30-12	139.53	17.25	122.28	0.3294	0.0067	9.44

### 3.5 The changes in colloid performance

**Fig. 6:**
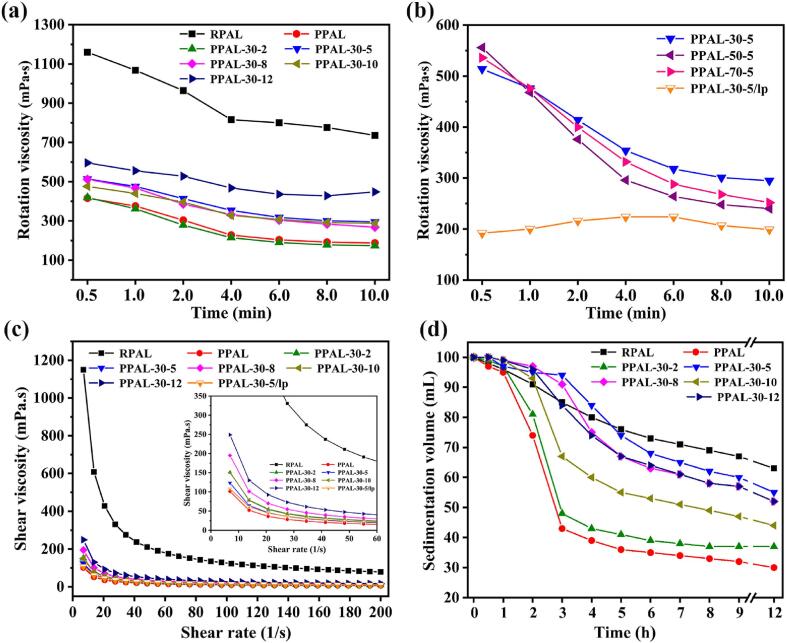
Variation of rotation viscosity (a, b), rheological viscosity (c), and sedimentation volume (d) of RPAL, PPAL, PPAL-30-*x* suspension.

As an important gel-forming mineral and viscosifying material, PAL with trioctahedral structure from the Mingguang mine has excellent colloidal and rheological properties even at low concentrations [48]. The full utilization of the intrinsic colloid performance of PAL is also closely related to the dispersion degree and colloidal stability of rod-like crystals in the medium [33]. Therefore, the apparent viscosity, sedimentation behavior, and rheological properties of RPAL, PPAL, and PPAL-30-*x* were systematically studied. As shown in Fig. 6a, the rotary viscosity of the RPAL aqueous suspensions gradually decreases with the increase of shear time, showing excellent shear-thinning thixotropy [51]. The rotary viscosity significantly reduces from 1160 mPa·s of RPAL to 416 mPa·s of PPAL after acid treatment, and then increased to 596 mPa·s for PPAL-30-12 after ultrasonic treatment. This phenomenon indicates that the changes in surface charge and dispersion degree of rod-like crystals directly affect the structure and stability of the constructed three-dimensional colloidal network [33]. In contrast to the increase in rotational viscosity of PAL suspension after high-pressure homogenization, the decrease in rotational viscosityis mainly attributed to the decrease in surface electronegativity after ultrasonic disaggregation [48]. It can be seen from Fig. 6b that the increase in the ultrasonic power has a greater effect on the rotational viscosity of the disaggregated powder than that of ultrasonic temperature in the sonochemical process. Because the increase in the ultrasonic temperature mainly affects the difficulty of cavitation events and collapse intensity by changing the vapor pressure and viscosity of liquid phase, while the increase in the ultrasonic power directly affects the number of cavitation events and the extent of acoustic streaming and turbulence [24]. The change of shear viscosity with the shear rate is similar to that of the rotation viscosity (Fig. 6c). All of the shearing rheological curves are not straight lines, indicating that the suspensions have a typical non-Newtonian fluid feature and shear-thinning behavior [51].

The colloidal stability of a uniform suspension can be intuitively evaluated by the sedimentation volume of the standing suspension [18]. As shown in Fig. 6d, the PPAL and PPAL-30-2 suspensions significantly settled by more than 50% at the initial 3 h. On the contrary, no sedimentation phenomenon is observed for the PPAL-30-5 and PPAL-30-8 suspensions, and their volumes remain at 94 and 91 mL, respectively, which is higher than that of the RPAL suspension (85 mL). After standing for 5 h, the volumes of other suspensions were less than 67 mL, while the volume of PPAL-30-5 suspension is up to 76 mL. It indicates that PPAL-30-5 exhibits the best suspension stability. Due to the low viscosity and weak surface electronegativity, PPAL-30-5 still exhibited similar suspension stability to RPAL after standing for 12 h, which was ultimately attributed to the improved dispersibility of rod crystal by ultrasonic disaggregation.

### 3.6 The application of PPAL-30-5 in drilling fluids as viscosifier

**Fig. 7:**
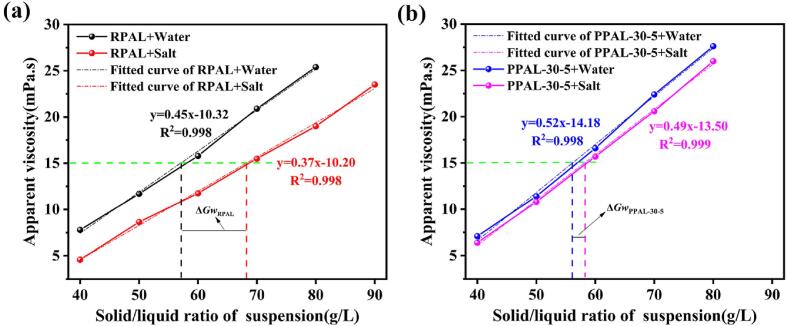
Variation of the pulping rate of RPAL (a) and PPAL-30-5 (b) after dispersions in deionized water and saturated sodium chloride solution (NaCl).

The previous research has established that both of the morphology and surface charge of the particles have a significant influence on the suitability as the drilling fluid, and the pulping rate under different liquid systems is one of the key technical indexes to evaluate the properties of the drilling fluid and the suitability of the materials [37]. The physicochemical properties of PAL before and after ultrasonic disaggregation can be well illustrated by RPAL and PPAL-30-5. Therefore, RPAL and PPAL-30-5 were taken as examples to test the pulping rate in the brine system and the pure water system. As shown in Fig. 7a, the minimum solid–liquid ratio (Gw) of *AV* = 15 mPa·s was 56.26 and 68.11 g/L when RPAL was dispersed in deionized water and saline solution, respectively. Therefore, the pulping rate of RPAL in the brine system (12.37 m^3^/t) significantly decreases compared with the pure water system (14.72 m^3^/t), because the addition of electrolytes reduces the energy barrier to Van der Waals attraction and electrostatic repulsion between the particles by compressing the electrical double layers and adsorbing ion in the Stern layer [52]. However, the Gw of PPAL-30-5 dispersed in deionized water and saline solution are 56.14 and 58.16 g/L, and the △*Gw*_PPAL-30-5_ (Gw_salt_- Gw_water_) is much smaller than △*Gw*_RPAl_ (Fig. 7b). The results suggest that PPAL-30-5 can maintain higher pulping rate in the brine and pure water systems, which is a more ideal viscosifier than RPAL for drilling fluids. This may be due to the highly dispersed rod crystals, excellent colloidal properties, and small particle size of PPAL-30-5, which allow for a denser and more stable network structure in the drilling fluid even at saturated salt content [52].

Table 2 lists the pulping rate of the drilling fluids prepared with different clay minerals. It is apparent that the pulping rate of different clay minerals from different regions varies greatly. By contrast, bentonite has a slightly lower pulping rate due to its natural layered structure and swelling properties [53], but PAL clays from Georgia and Florida in the United States (denoted as Na-PAL(G) and Na-PAL(F)) have a higher pulping rate (>20 m^3^/t), and the pulping rate of PAL from Sacalum in Mexico (denoted as Na-PAL(S)) is up to 17.81 m^3^/t. In addition to the effect of Na-saturation treatment, the well-dispersed nanorod crystals (length of 816 ± 233 nm and width of 29 ± 7 nm) may be also a key factor for the high pulping rate [54]. Although PPAL-30-5 has a moderate pulping rate of 14.45 m^3^/t in the saturated brine system and 14.96 m^3^/t in the water system, PPAL-30-5 can be classified as type 1 palygorskite (the pulping rate is higher than 12.5 m^3^/t) in both saturated and salt-free conditions [37]. In other words, PPAL-30-5 is a suitable drilling fluid additive due to its high pulping rate and excellent salt tolerance.

**Table 2:** Comparison of the pulping rate of drilling fluids prepared with PAL and bentonite.

Slurry samples	Gw/(g/L)	pulping rate/(m^3^/t)	References
Na-PAL(G) + Water	35.00	23.81	[54]
Na-PAL (F) + Water	38.00	21.96	[54]
Na-PAL (S) + Water	47.00	17.81	[54]
Bentonite (XJ) + Water	95.60	8.91	[53]
Bentonite (LN) + Water	138.20	6.25	[53]
Bentonite (NM) + Water	63.02	13.36	[53]
Bentonite (HB) + Water	55.10	15.24	[58]
RPAL + Water	56.26	14.72	This work
PPAL-30-5 + Water	56.11	14.96	This work
RPAL + Salt	68.11	12.37	This work
PPAL-30-5 + Salt	58.16	14.45	This work
PAL (B.V) + Salt	–	7.20	[37]
PAL (W.S.P) + Salt	–	13.00	[37]
PAL (D.S.P) + Salt	–	16.40	[37]

## 4 Conclusions

In this study, a feasible industrial disaggregation technology centered on ultrasonic treatment was developed for the large-scale production of PAL individual nanorods on the pilot production line. The results showed that the disaggregation of PAL crystal bundles can be successfully achieved by ultrasonic treatment without damaging the structure and length of the rod crystals. After ultrasonic treatment at 20 kHz, 2000 W, and 30 °C for 5 min, the dispersion, suspension capacity, and colloid performance of the treated PAL were significantly improved. More importantly, the pulping rate in saturated saline of PPAL-30-5 (14.45 m^3^/t) was significantly improved compared with RPAL (12.37 m^3^/t). In conclusion, the non-destructive and efficient disaggregation of PAL crystal bundles by the ultrasonic process was realized in the industrial process, and the disaggregated products can be well applied to drilling operations in a high salinity environment, which helps to expand the potential utilization of PAL.

## Declaration of Competing Interest

The authors declare that they have no known competing financial interests or personal relationships that could have appeared to influence the work reported in this paper.

## Data Availability

Data will be made available on request.
