# Supplementary material for: Scale-up disaggregation of palygorskite crystal bundles via ultrasonic process for using as potential drilling fluid
Source: Ultrason Sonochem. 2022 Aug 19;89:106128. doi: 10.1016/j.ultsonch.2022.106128 (PMC9428861; doi:10.1016/j.ultsonch.2022.106128)
Supplement: Supplementary data 1 [file mmc1.doc]

**Scale-up disaggregation of palygorskite crystal bundles via ultrasonic process for using as potential drilling fluid**

Jiang Xu ^a^, Wenbo Wang^b^, Yushen Lu ^a^, Hong Zhang ^a,c^, Yuru Kang ^a^, Bin Mu ^a^, Ye Qian^d^, Aiqin Wang a,[[1]](#footnote-2)*

^a^Key Laboratory of Clay Mineral Applied Research of Gansu Province, Center of Eco-material and Green Chemistry, Lanzhou Institute of Chemical Physics, Chinese Academy of Sciences, Lanzhou 730000, P.R. China

^b^College of Chemistry and Chemical Engineering, Inner Mongolia University, Hohhot, 010021, P.R. China

^c^Center of Materials Science and Optoelectronics Engineering, University of Chinese Academy of Sciences, Beijing 100049, P.R. China

^d^Department of Civil Engineering, The University of Hong Kong, Pokfulam Road, Hong Kong, China


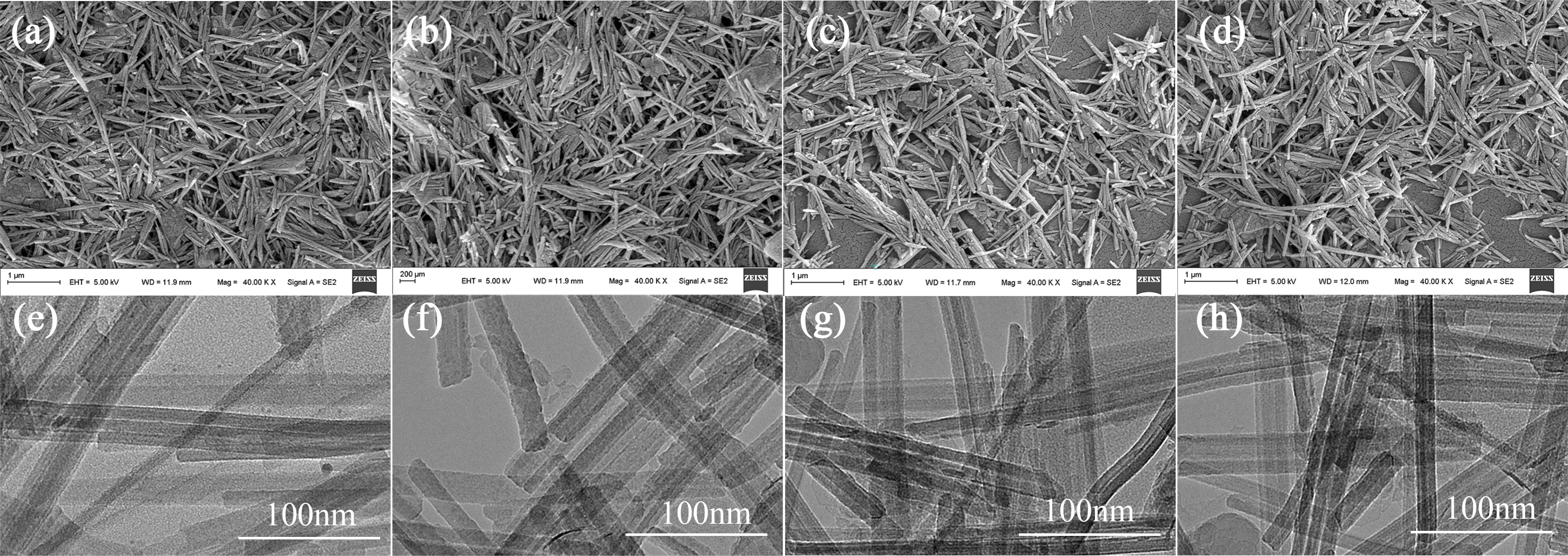


**Fig. S1** SEM and TEM images of PPAL-30-8 (a, e), PPAL-30-10 (b, f), PPAL-50-5 (c, g) and PPAL-70-5 (d, h)


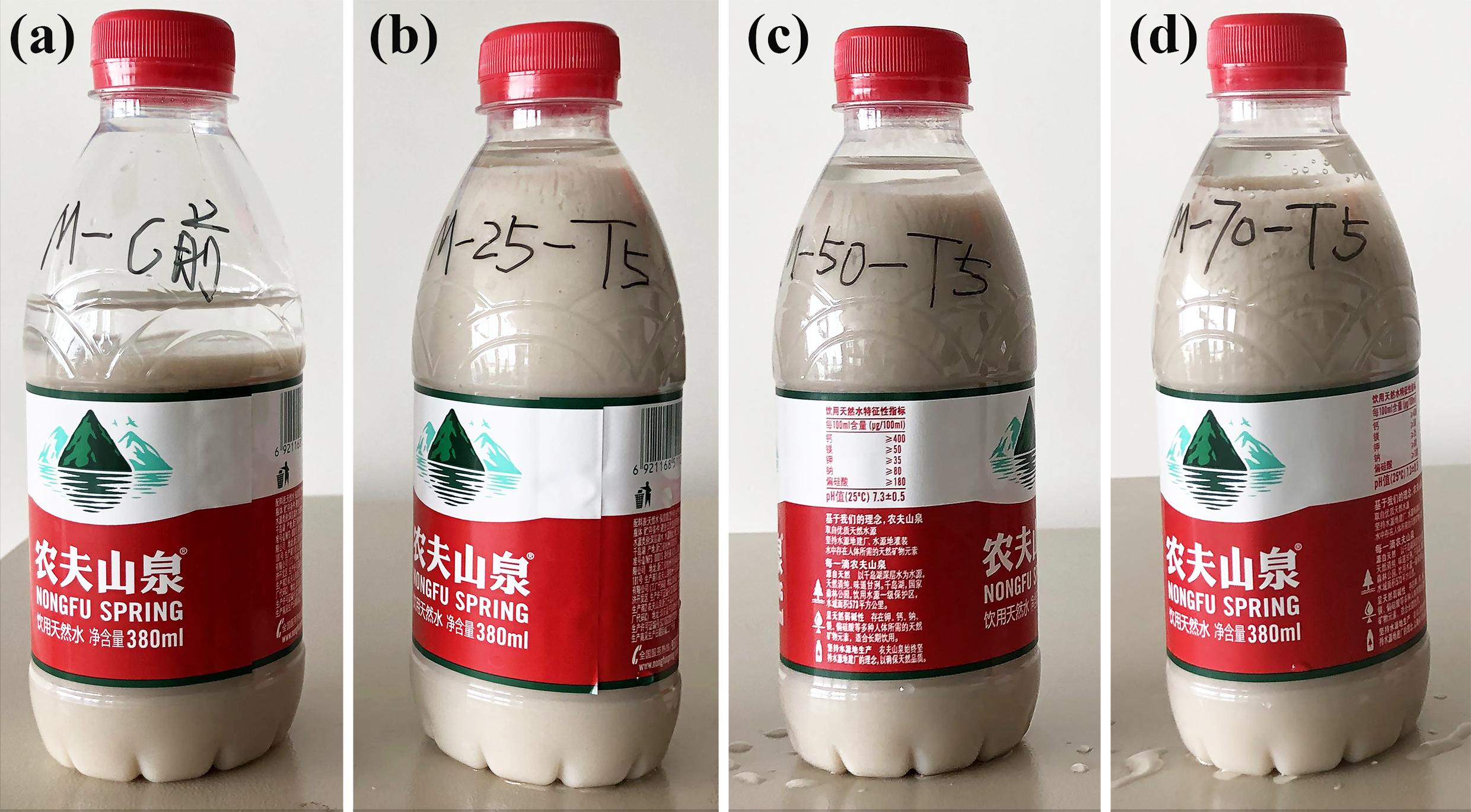


**Fig.S2** Digital photo of PPAL (a), PPAL-30-5 (b), PPAL-50-5 (c) and PPAL-70-5 (d) suspension

**
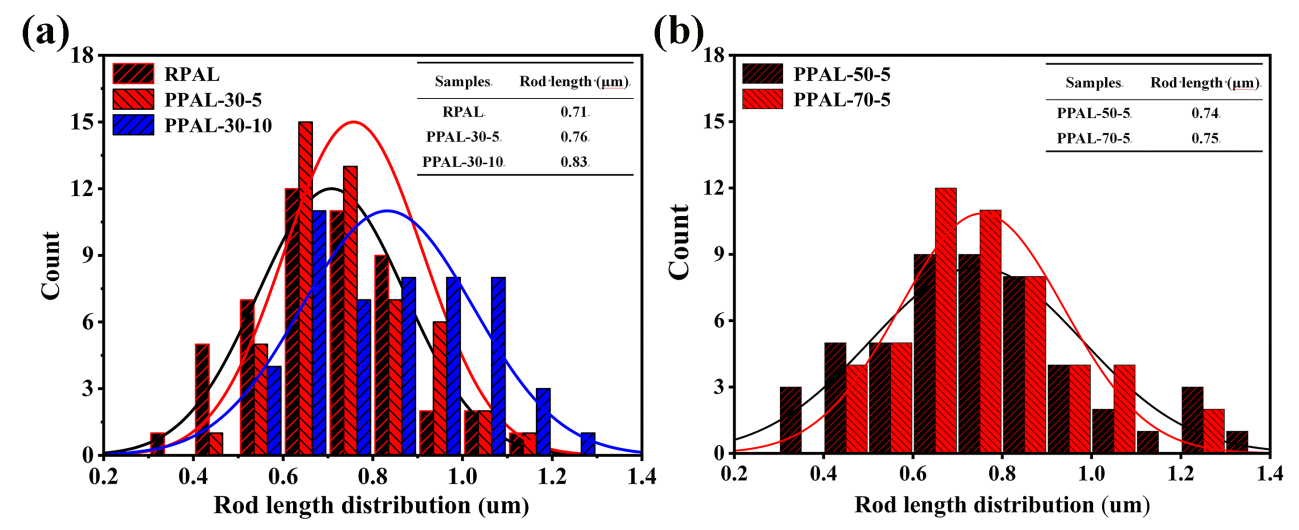
**

**Fig. S3** Rod length distribution of RPAL and PPAL-30-*x* (a),

PPAL-50-5 and PPAL-70-5 (b)


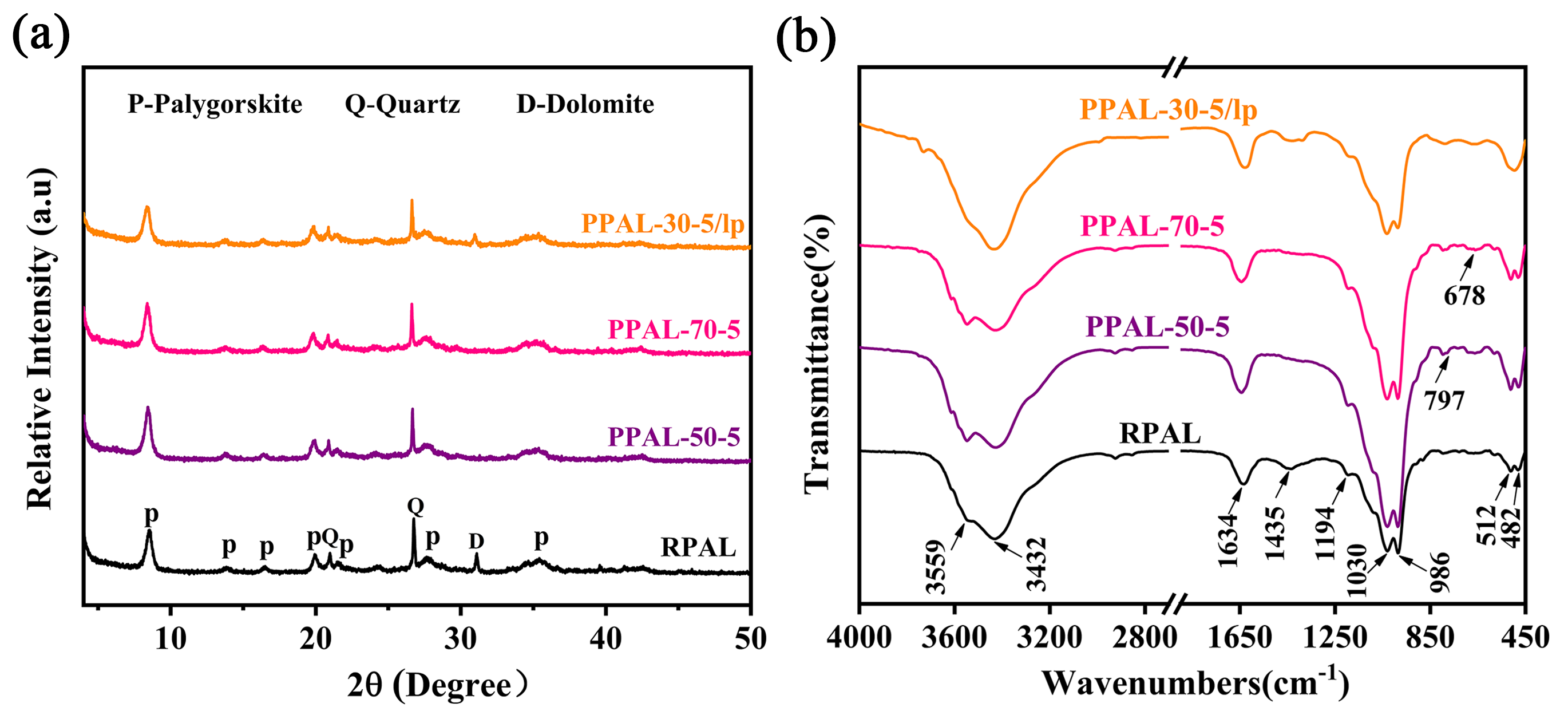


**Fig.S4** XRD patterns (a) and FTIR spectra (b) of PPAL-50-5, PPAL-70-5 and PPAL-30-5/lp

**Table S1** Chemical composition of natural RPAL and the treated samples

| Samples |  | Mass (%) | | | | | | |
| --- | --- | --- | --- | --- | --- | --- | --- | --- |
|  | SiO_2_ | | Al_2_O_3_ | MgO | Fe_2_O_3_ | K_2_O | CaO | SO_3_ |
| RPAL | 59.61 | | 9.83 | 10.98 | 5.19 | 1.08 | 2.61 | 169.70(ppm) |
| PPAL | 60. 83 | | 10.73 | 9.70 | 5.71 | 1.18 | 0.49 | 0.79 |
| PPAL-30-2 | 60.81 | | 10.66 | 9.60 | 5.75 | 1.19 | 0.54 | 0.90 |
| PPAL-30-5 | 60.82 | | 10.61 | 9.55 | 5.74 | 1.20 | 0.56 | 0.94 |
| PPAL-30-8 | 60.70 | | 10.66 | 9.64 | 5.75 | 1.19 | 0.55 | 0.95 |
| PPAL-30-10 | 60.62 | | 10.63 | 9.63 | 5.73 | 1.18 | 0.58 | 1.06 |
| PPAL-30-12 | 61.20 | | 8.24 | 9.98 | 6.01 | 1.22 | 0.79 | 1.82 |
| PPAL-50-5 | 62.13 | | 8.18 | 9.89 | 6.01 | 1.25 | 0.68 | 1.26 |
| PPAL-70-5 | 61.80 | | 8.25 | 9.82 | 5.95 | 1.24 | 0.81 | 1.53 |
| PPAL-30-5/lp | 64.68 | | 12.01 | 10.88 | 7.22 | 1.67 | 2.25 | 0.17 |

**Table S2** Particle size classification of RPAL and the treated samples

| Samples | Particle diameter distribution | | |
| --- | --- | --- | --- |
|  | D_10_ (μm) | D_50_ (μm) | D_90_ (μm) |
| RPAL | 10.68 ± 0.53 | 91.70 ± 2.60 | 297.33 ± 4.93 |
| PPAL | 3.37 ± 0.03 | 21.75 ± 0.35 | 94.05 ± 0.25 |
| PPAL-30-2 | 4.22 ± 0.03 | 29.43 ± 0.60 | 97.80 ± 0.10 |
| PPAL-30-5 | 4.29 ± 0.03 | 30.00 ± 0.85 | 93.23 ± 1.65 |
| PPAL-30-8 | 4.41 ± 0.06 | 32.83 ± 1.36 | 102.67 ± 2.52 |
| PPAL-30-10 | 4.34 ± 0.05 | 29.70 ± 1.41 | 99.90 ± 1.56 |
| PPAL-30-12 | 4.35 ± 0.08 | 28.07 ± 1.38 | 97.47 ± 2.19 |
| PPAL-50-5 | 4.30 ± 0.01 | 30.65 ± 0.49 | 97.30 ± 0.14 |
| PPAL-70-5 | 3.97 ± 0.08 | 25.07 ± 1.67 | 95.77 ± 3.50 |

1. ^^Corresponding author at: Key Laboratory of Clay Mineral Applied Research of Gansu Province, Center of Eco-material and Green Chemistry, Lanzhou Institute of Chemical Physics, Chinese Academy of Sciences, Lanzhou 730000, China. *E-mail address: aqwang@licp.cas.cn* (A.Q. Wang) [↑](#footnote-ref-2)
